# Towards Greater Collaboration in Educational Neuroscience: Perspectives From the 2018 Earli‐SIG22 Conference

**DOI:** 10.1111/mbe.12250

**Published:** 2020-06-18

**Authors:** Nienke van Atteveldt, Sabine Peters, Bert De Smedt, Iroise Dumontheil

**Affiliations:** ^1^ Faculty of Behavioural and Movement Sciences, Section of Clinical Developmental Psychology and Institute Learn! Vrije Universiteit Amsterdam Netherlands; ^2^ Department of Developmental and Educational Psychology, Institute of Psychology and Leiden Institute for Brain and Cognition Leiden University Netherlands; ^3^ Faculty of Psychology and Educational Sciences, Parenting and Special Education Research Unit University of Leuven Belgium; ^4^ Department of Psychological Sciences, Centre for Brain and Cognitive Development Birkbeck, University of London UK; ^5^ Centre for Educational Neuroscience University of London UK

## Abstract

The special issue resulting from the 2018 Earli‐SIG22 conference reflects the current state of the field, the diversity of methods, the persevering limitations and promising directions towards solutions. About half of the empirical papers in this special issue that consist of three parts, uses behavioral, self‐report or qualitative measures to understand the “mind” level of *Mind, Brain, and Education*. The other half investigates the “brain” level, using neuroimaging but also genetics or eye‐tracking to gain access to the wider range of biological substrates of learning and cognition. These biological studies mostly have added value by refining psychological theories, such that these inspire new hypotheses to test in the field, to ultimately better inform teaching. Importantly, the special issue presents several approaches to more intensive, bi‐directional and systematic practice‐research collaborations to better connect the “mind” and “brain” levels to education, and to equip researchers to realize such collaborations successfully in the future.

After more than a decade of *Mind, Brain, and Education* (*MBE*) research, the 2018 Earli‐SIG22 conference provided a great opportunity to gauge the current state of the field, its persevering limitations, and its most promising directions for future research. The organizers took a very conscious approach to arranging the meeting, based on feedback from the 2016 IMBES meeting (Brookman‐Byrne & Commissar, 2019, pt. I). This resulted in more room for the perspective of practitioners and more interaction between researchers and practitioners. A new format was introduced: An Open Space event, which contributed to more collaboration among researchers. Delegates were completely free to create their own sessions and subsequently brainstorm in small groups. This format appeared very promising for encouraging collaboration, as demonstrated for instance by Hobbiss et al. (2019, pt. II), a paper that resulted from a collaboration initiated during the Open Space event. Brookman‐Byrne and Commissar (2019, pt. I) further report the results of an exit survey among conference delegates, evaluating their vision and intentions for research and translation, and the support they need to move forward. New ideas and avenues for strengthening research‐teacher collaborations were proposed, such as inclusion of learners in research design, open resources for teacher training in neuroscience, and mentoring networks for teachers and researchers.

The articles published in the current special issue that was published in three parts resonate with these survey results. They illustrate the diversity of methods and perspectives in the field. About half of the empirical papers in this special issue use behavioral, self‐report or qualitative measures to understand the “mind” level of *MBE*. Many of the results have potentially important implications for educational practice, such as science and mathematics teaching. However, when translating *MBE* research to practice, we should be careful to avoid creating new misunderstandings or so‐called neuromyths, a topic that was addressed in two empirical studies in this issue.

The other half of the empirical papers use biological measures to investigate the “brain” level of *MBE*, using functional magnetic resonance imaging (fMRI), functional near‐infrared spectroscopy (fNIRS), or electroencephalography (EEG). Some studies also included genetics or eye‐tracking, to gain access to the wider range of biological substrates of learning and cognition. The studies add to our understanding of the biological processes related to cognitive functions. They complement and deepen the implications provided by the behavioral studies, for example, related to science and mathematics education or effective feedback.

This special issue also includes two reviews that highlight the gaps in *which processes* are investigated in *MBE* studies, and the lack of conceptual clarity on the *level* at which these processes are studied (shallow vs. deep). Finally, in line with Brookman‐Byrne and Commissar (2019, pt. I), the special issue puts forward several approaches to more intensive, bi‐directional and systematic practice‐research collaborations, and reports experiences with the related practical challenges and ways to overcome them. These articles address how the “mind” and “brain” levels of research can be more optimally linked to the “education” level.

The following sections summarize the main findings of the articles in this special issue and their implications for the *MBE* community and educational practice. We first discuss the empirical studies and reviews, and end with the suggested approaches to carry out research‐practice collaborative research.

## UNRAVELING THE “MIND” ASPECTS OF LEARNING: BEHAVIORAL STUDIES

O'Connor, Morsanyi, and McCormack (2019, pt. I) examined cognitive predictors of children's early mathematical skills at the age of 4 and 6, right before the start of formal education. They showed that particularly nonnumerical ordering skills were stable and reliable predictors of early formal mathematical skills. They suggest that not only numerical but also nonnumerical cognitive skills form the basis of formal mathematics learning and consequently, both sets of skills should be considered when thinking about prediction and intervention.

Not only child‐specific characteristics, but also the social environment is important when learning math. “Math‐talk” is an important aspect of early math development. Von Spreckelsen et al.' (2019, pt. II) qualitative results show that early educator's math‐talk was biased towards using mostly counting. Quantitative analyses showed that the variety of math talk (including counting, but also size comparisons, place‐value language) predicted later cardinality skills. Enriching educators' mathematical language may be an effective way to improve children's early math outcomes.

Dündar‐Coecke and Tolmie (2019, pt. III) investigated the role of verbal and nonverbal ability in causal reasoning in a large group of primary school children. They found that verbal and nonverbal skills were dissociable contributors to children's causal reasoning. Their findings have implications for science education, where the current focus is mostly on verbal instruction.

Using another approach to study science education, Morris, Farran, and Dumontheil (2019, pt. II) found that field independence, which reflects the ability to resist influence from the context (the forest) when identifying embedded shapes (the trees), uniquely correlates with performance on tests of mathematical reasoning and science in primary school. These results indicate that mathematics and science problems often require children to separate parts and whole, whether visually or conceptually. It therefore may be beneficial to teach children to orient their attention towards key elements (e.g., an operation symbol) and ignore distracting information. It also indicates that the way mathematics and science problems are presented will affect performance.

Although educators are often highly motivated to translate *MBE* research to practice, it is crucial to ensure that such translations do not result in new neuromyths. Two articles in this special issue provide possibilities for investigating and reducing neuromyth belief in teachers. Tovazzi, Giovanni, and Basso (2020, pt. III) designed and tested a new type of questionnaire to capture neuromyth belief among teachers, by integrating myths within scenarios of realistic classroom situations. In this new questionnaire, neuromyth belief was significantly lower than in the more standard neuromyth questionnaire. This may suggests that teachers' adhesion to neuromyths in realistic situations does not necessarily match their more explicit beliefs. McMahon, Yeh, and Etchells (2019, pt. II) worked with tutors and trainee teachers on adapting an initial teacher‐training program to reduce trainee's beliefs in neuromyths. Questionnaire data collected 8 months apart in 130 teacher trainees suggested that the trainees' beliefs in neuromyths had become unsettled. Trainees were more critical about applying *MBE*‐research in educational contexts, however a number of neuromyths persisted. Although there was no control group, this work is promising. Importantly, it reveals the constraints imposed by the short duration of initial teacher training and the resistance of neuromyths to short interventions.

## UNRAVELING THE BIOLOGICAL SUBSTRATES OF LEARNING: NEUROIMAGING, GENETIC, AND PHYSIOLOGICAL STUDIES

Two fMRI‐studies are incorporated in this special issue. Brookman‐Byrne, Mareschal, Tolmie, and Dumontheil (2019, pt. I) investigated in 11–15‐year‐old adolescents the associations between relational reasoning, that is, the ability to detect meaningful patterns, and their ability to solve math and science problems. Individual differences in verbal analogical reasoning and nonverbal matrix relational reasoning were correlated with performance on mathematics and science problems as well as with their patterns of neural activation. The associations with mathematics achievement disappeared when individual differences in executive functions and verbal intelligence quotient were taken into account, yet the associations with science learning remained. These results highlight the potential specific role of analogical and relational reasoning in science, which may support the understanding of new, often abstract, concepts, through analogies (Vendetti, Matlen, Richland, & Bunge, 2015).

van der Aar, Crone, and Peters (2019, pt. I) used fMRI and behavioral measures to investigate the role of self‐esteem and associated neural activity on making future‐oriented study choices. Many adolescents struggle with choosing a well‐suited educational career path after high‐school. This study revealed that adolescents who experience these difficulties have lower levels of self‐esteem and self‐concept clarity. Moreover, these adolescents had less activity in the medial prefrontal cortex during self‐related processing. A potential implication is that fostering self‐esteem and self‐knowledge deserves more attention towards the end of high school. Many schools currently take the approach of overloading teenagers with information about potential career options, rather than encouraging them to reflect upon their own motives, interests and skills.

Soltanlou et al. (2019, pt. II) used fNIRS to investigate whether letters and numbers recruit distinct or shared neural networks. They examined this question in fifth and sixth grade children, who were approximately 11 and 12 years old, respectively. Different from many brain imaging studies in which participants have to respond via key presses, they investigated the processing of numbers and letters in a more ecologically valid way, as they asked children to write down numbers and letters, as they would be doing in the classroom, while their brain activity was recorded via fNIRS. The findings revealed similar brain activation patterns for numbers and letters. This suggests, at least at this age, shared neural networks for symbols that are learned via education.

Feedback processing is a good example of where cognitive neuroscience methods have a clear added value. Behaviorally, one can measure only what happens after a student receives feedback (e.g., the next response), but not what happens *during* receiving the feedback. Dainton, Winstone, Klaver, and Opitz (2019, pt. III) used EEG to relate processing of different types of feedback to accuracy and rate of learning in university students performing a category learning task. They manipulated whether the feedback was easy or hard to decode, and of low versus high utility (i.e., providing information to learn from). Behaviorally, high utility feedback resulted in higher accuracy, but only for feedback that was easy to decode. The authors suggest that the benefits of more useful feedback only apply to easy‐to‐decode information, but when information is hard‐to‐decode, utility is less pertinent to learning. Their results therefore imply that feedback should be easy to understand and highly applicable. The EEG data revealed larger feedback‐related negativity for high‐utility feedback, indicating stronger processing as the underlying mechanism for better learning. The EEG data added the insight that this increased feedback processing was specific for trials following negative feedback.

Cognitive neuroscience measures can also be used to complement behavioral and cognitive measures during the evaluation of school‐based interventions (e.g., Neville et al., 2013). Wu and Kim (2019, pt. II) conducted a 10‐week story‐based tablet game aiming to enhance 3–6‐year‐olds developing empathy skills. They compared 14 children who took the 30 sessions of 10+ min of individualized training to 12 children in a passive control condition. Event‐related potentials (ERPs) were used as outcome measures. The intervention group showed increased attention to others' feelings, as reported by teachers, and changes in the P2 ERP component, thought to reflect greater attention to anti‐social/harming scenarios. Crucially, training‐related changes in behavioral and neural measures correlated with each other. However, more research is needed to replicate these results in larger samples and with an active control group.

Galili, Babai, and Stavy (2020, pt. III) used eye‐tracking to investigate attention to irrelevant salient variables in the Comparison of Perimeters Task. This task has previously been shown to elicit errors, for example, if two shapes have the same surface area, their perimeters are concluded to be the same. The congruency of differences in surface area and differences in perimeters was linked with specific patterns of eye movements, suggesting that surface area may be automatically and unintentionally processed. More research is needed to explore attention to irrelevant salient information and to test whether the pattern observed in this study is observed in more varied math and science problems with perceptually misleading information.

Genome‐wide association studies (GWAS) offer the potential to study the underlying genetic contributions to learning. Donati, Dumontheil, and Meaburn (2019, pt. III) analyzed data from a large longitudinal cohort of adolescents to investigate the genetic contribution to executive function and their genetic relationship with academic attainment and intelligence. They found common genetic contributions to working memory and processing speed, but to inhibitory control. Working memory shared genetic effects with academic attainment and intelligence, and it showed a negative genetic correlation with attention deficit and hyperactivity disorder. These data indicate that the genetic effects underlying working memory are pleiotropic and that they also affect lifespan intelligence and academic attainment. More modest genetic overlaps between processing speed and intelligence were observed, which suggests that distinct biological pathways contribute to processing speed.

## REVIEWING THE EMPIRICAL EVIDENCE: SHALLOW VERSUS DEEP PROCESSING AND CONCEPTUAL CHANGE

Two review papers were included in this special issue. A key question in education is how shallow versus deep processing contributes to learning. This is especially important because many teachers aspire to encourage “deep” processing of their lesson material. Catrysse, Gijbels, and Donche (2019, pt. I) conducted a systematic review, which included 25 papers, to examine how levels of processing are studied with fMRI. The findings reveal that fMRI research on levels of processing uses only highly controlled conditions with decontextualized and simplified stimuli. There is also a lack of conceptual clarity: In half of the cases, no theoretical framework or definition of levels of processing was explicitly mentioned. To further our understanding of how the brain processes information in a deep way, a more clearly defined conceptual framework is needed. This will help to understand the brain mechanisms that support the deep processing of information, which could in turn inform teaching to develop the best methods to stimulate deep processing of information.

Vaughn, Brown, and Johnson (2020, pt. III) emphasize in their review that many studies in the field of *MBE* have focused on language and mathematics learning, yet that only a few have applied this framework to conceptual change and science learning. Reviewing the existing body of data, Vaughn et al. indicate that *MBE*‐studies in the field of conceptual change and science learning are starting to reveal important new insights related to individuals' misconceptions as well as the roles of error detection and executive function in conceptual change. Acknowledging the limitations of brain imaging to investigate these more complex cognitive abilities, Vaughn et al. present some future directions for the study of conceptual change through the lens of *MBE*, highlighting the critical need to form collaborative partnerships across different disciplines.

## RESEARCH‐PRACTICE COLLABORATION AS A NEW STARTING POINT FOR *MBE* RESEARCH

In addition to interdisciplinary collaborations across researchers, many members of the *MBE* community stress the importance of more intensive, bidirectional collaborations between researchers and practitioners from the start of a research project (Brookman‐Byrne & Commissar, 2019, pt. I). Despite these good intentions this is often not straightforward to realize. Several studies in this special issue provide researchers with examples of how to foster more intensive collaborations with educational practitioners.

Three papers in this special issue took a practical approach in designing and performing research together with educators. Van Atteveldt, Tijsma, Janssen, and Kuppers (2019, pt. II) propose a responsible research and innovation (RRI) framework, which has been used in other fields already, to improve the alignment between research, educational practice, and other societal stakeholders. They apply this framework in a case study for developing an intervention to improve children's sense of agency in learning with neurofeedback. Using RRI, it became clear that societal stakeholders (teenagers, parents, and teachers) had different expectations about this intervention than researchers. This shows how RRI before starting an intervention can be useful to take these multiple perspectives into account at an early stage. This might minimize potential negative impacts and miscommunication.

Massonnié, Frasseto, Mareschal, and Kirkham (2020, pt. III) examined teacher–researcher collaboration for investigating the effect of noise reduction in elementary classrooms. They observed this collaboration during the following stages of the research: selecting research questions, planning interventions, obtaining ethical approval, recruiting schools and collecting data. For each step, their paper provides highly useful suggestions and concrete examples for future collaborations.

A large‐scale approach was taken by Churches et al. (2020, pt. III). They initiated and reported on 34 randomized‐controlled‐trials (RCTs) based on science of learning‐translated pedagogy. Teachers designed the RCTs in collaboration with researchers, focusing on topics such as attention, memory and spaced learning. A meta‐analytic approach was used to examine the overall effect of these 34 RCTs that were run over the diverse range of topics. This analysis revealed that overall, teacher‐led RCTs resulted in positive short‐term effects. The authors highlight that multiple planned teacher‐led RCTs and replications as well as meta‐analytic analytic approaches that combine the outcomes of different studies, show promising potential as an evaluation tool.

Hobbiss et al. (2019, pt. II) note that while the need for collaboration between researchers and practitioners has been recognized and emphasized repeatedly in the last decade, relatively few of such collaborations have actually been realized. They report a SWOT analysis of such transdisciplinary partnerships in the field of *MBE*, based on literature. They use this analysis to inform the development of an international web platform to broker relationships between researchers and teachers (based on interests and location). This team and their ideas for this project were formed during the Open Space event during the SIG22 conference, indicating that such meetings can play an important role in bringing multidisciplinary teams, including practitioners, together.

## CONCLUSIONS

Behavioral studies increase our understanding of development, learning, and teaching. Neuroimaging studies can complement behavioral data and improve our understanding of mechanisms through more implicit, “online” measures that are closer to the neural systems at play. Thomas, Ansari, and Knowland (2019) recently provided an excellent overview of how neuroscience and education can interact. They argue that one of the key ways neuroscience can be useful to education is by providing constraints on psychological theories based on how the brain works. The empirical articles in this special issue mostly correspond to what Thomas et al. (2019) refer to as an indirect interaction between neuroscience and education, as these studies refine psychological theories, such that these theories can better inform teaching.

There are several examples of this indirect potential benefit for a diverse range of research topics in this special issue. The study by Dainton et al. (2019, pt III) on feedback‐processing provides important hints about which types of feedback are more useful in the learning process and why. Feedback that was highly applicable and useful was processed in a different way with higher FRN‐amplitudes, suggesting “stronger” processing which may enable better learning results. This is an example of how neuroscience methods provide additional information over behavioral measures. This information can be used to develop new hypotheses, which can subsequently be tested behaviorally. Similarly, Galili et al. (2020, pt. III) show how an “online” psychophysiological measure can provide insights into the processes underlying certain behaviors. In their case, the eye tracking data did not show the same effects as the behavioral data when inspecting what happens during the processing of irrelevant salient information. In addition, Wu and Kim (2019, pt. II) showed that EEG also provided complementary insights over behavior alone. In their study, EEG was used to obtain an online measure of attention to social situations, to complement teacher reports on increased empathy. The EEG findings suggested greater attention to anti‐social scenarios after an intervention.

While many studies in the *MBE*‐field focus on the cognitive factors underlying learning, there is a gap of knowledge in more socio‐affective aspects of learning. Van der Aar et al.'s (2019, pt. I) study on educational/career‐choices showed that fostering self‐esteem and self‐knowledge may be important for this process and deserves more attention towards the end of high school. The medial prefrontal cortex played an important role, which is relevant because this is a key region of the social brain, which shows protracted development until the early twenties (Mills, Lalonde, Clasen, Giedd, & Blakemore, 2014). This is an example of neuroscience research that might inform current school practices.

A challenge in designing neuroimaging studies in the *MBE*‐field deals with the issue of ecological validity. van Atteveldt, van Kesteren, Braams, and Krabbendam (2018) recently suggested several directions for improving ecological validity of developmental neuroimaging studies. One of these directions is by using less static neuroimaging equipment. An example from this special issue is the fNIRS study by Soltanlou et al. (2019, pt. II). fNIRS allows participants more movement freedom compared to fMRI, enabling the use of less simplistic and less controlled tasks.

Another promising direction for improving ecological validity is a more intense collaboration with teachers, which might ensure finding a better balance between experimentally controlled vs. ecologically valid studies. Van Atteveldt et al. (2019, pt. II) and Hobbiss et al. (2019, pt. II) provide important examples of *how* the field can move forward with increased collaboration between researchers, teachers, parents, and children. It is important to avoid the pitfall of lecturing teachers about what they should be doing in the classroom, when teachers have little or no voice in the research agenda of educational neuroscience (Thomas et al., 2019). The same holds for parents and children. We contend that such collaborative approaches can also help teachers, parents and children develop realistic expectations from neuroscience research. It will help to prevent the emergence of new neuromyths.

## Conflict of interest

None declared.

## References

[mbe12250-bib-0001] Brookman‐Byrne, A. , & Commissar, L. (2019). Future avenues for educational neuroscience from the perspective of EARLI SIG 22 conference attendees. Mind, Brain, and Education, 13(3), 176–183. 10.1111/mbe.12211 PMC6774272 31598131

[mbe12250-bib-0002] Brookman‐Byrne, A. , Mareschal, D. , Tolmie, A. K. , & Dumontheil, I. (2019). The unique contributions of verbal analogical reasoning and nonverbal matrix reasoning to science and maths problem‐solving in adolescence. Mind, Brain, and Education, 13(3), 211–223. 10.1111/mbe.12212 PMC7189624 32362934

[mbe12250-bib-0003] Catrysse, L. , Gijbels, D. , & Donche, V. (2019). A systematic review on the conceptualization and operationalization of students' levels of processing in functional magnetic resonance imaging studies. Mind, Brain, and Education, 13(3), 198–210.

[mbe12250-bib-0004] Churches, R. , Dommett, E. , Devonshire, I. M. , Hall, R. , Higgins, S. , & Korin, A. (2020). Translating laboratory evidence into classroom practice with teacher‐led randomised controlled trial—A perspective and meta‐analysis. Mind, Brain and Education. 10.1111/mbe.12243

[mbe12250-bib-0005] Dainton, C. , Winstone, N. , Klaver, P. , & Opitz, B. (2019). Utility of feedback has a greater impact on learning than ease of decoding. Mind, Brain, and Education, 14(2), 139–145. 10.1111/mbe.12227

[mbe12250-bib-0006] Donati, G. , Dumontheil, I. , & Meaburn, E. L. (2019). Genome‐wide association study of latent cognitive measures in adolescence: Genetic overlap with intelligence and education. Mind, Brain, and Education, 13(3), 224–233. 10.1111/mbe.12198 PMC6771723 31598132

[mbe12250-bib-0007] Dündar‐Coecke, S. , & Tolmie, A. (2019). Nonverbal ability and scientific vocabulary predict children's causal reasoning in science better than generic language. Mind, Brain, and Education, 14(2), 130–138. 10.1111/mbe.12226

[mbe12250-bib-0008] Galili, H. , Babai, R. , & Stavy, R. (2020). Intuitive interference in geometry: An eye‐tracking study. Mind, Brain, and Education, 14(2), 155–166. 10.1111/mbe.12231

[mbe12250-bib-0009] Hobbiss, M. H. , Massonnié, J. , Tokuhama‐Espinosa, T. , Gittner, A. , de Sousa Lemos, M. A. , Tovazzi, A. , … Gous, I. (2019). “UNIFIED”: Bridging the researcher–practitioner divide in mind, brain, and education. Mind, Brain, and Education, 13(4), 298–312.

[mbe12250-bib-0010] Massonnié, J. , Frasseto, P. , Mareschal, D. , & Kirkham, N. Z. (2020). Scientific collaboration with educators: Practical insights from an in‐class noise‐reduction intervention. Mind, Brain, and Education. 10.1111/mbe.12240

[mbe12250-bib-0011] McMahon, K. , Yeh, C. S. H. , & Etchells, P. J. (2019). The impact of a modified initial teacher education on challenging trainees' understanding of neuromyths. Mind, Brain, and Education, 13(4), 288–297.

[mbe12250-bib-0012] Mills, K. L. , Lalonde, F. , Clasen, L. S. , Giedd, J. N. , & Blakemore, S.‐J. (2014). Developmental changes in the structure of the social brain in late childhood and adolescence. Social Cognitive and Affective Neuroscience, 9(1), 123–131. 10.1093/scan/nss113 23051898 PMC3871734

[mbe12250-bib-0013] Morris, S. , Farran, E. K. , & Dumontheil, I. (2019). Field Independence associates with mathematics and science performance in 5‐to 10‐year‐olds after accounting for domain‐general factors. Mind, Brain, and Education, 13(4), 268–278.

[mbe12250-bib-0014] Neville, H. J. , Stevens, C. , Pakulak, E. , Bell, T. A. , Fanning, J. , Klein, S. , & Isbell, E. (2013). Family‐based training program improves brain function, cognition, and behavior in lower socioeconomic status preschoolers. Proceedings of the National Academy of Sciences of the United States of America, 110(29), 12138–12143. 10.1073/pnas.1304437110 23818591 PMC3718115

[mbe12250-bib-0015] O'Connor, P. A. , Morsanyi, K. , & McCormack, T. (2019). The stability of individual differences in basic mathematics‐related skills in young children at the start of formal education. Mind, Brain, and Education, 13(3), 234–244.

[mbe12250-bib-0017] Soltanlou, M. , Coldea, A. , Artemenko, C. , Ehlis, A. C. , Fallgatter, A. J. , Nuerk, H. C. , & Dresler, T. (2019). No difference in the neural underpinnings of number and letter copying in children: Bayesian analysis of functional near‐infrared spectroscopy data. Mind, Brain, and Education, 13(4), 313–325.

[mbe12250-bib-0018] Thomas, M. , Ansari, A. , & Knowland, V. (2019). Annual research review: Educational neuroscience: Progress and prospects. Journal of Child Psychology and Psychiatry, 60(4), 477–492. 30345518 10.1111/jcpp.12973 PMC6487963

[mbe12250-bib-0019] Tovazzi, A. , Giovanni, S. , & Basso, D. (2020). A new method for evaluating knowledge, beliefs and neuromyths about the mind and brain among Italian teachers. Mind, Brain, and Education.

[mbe12250-bib-0020] van Atteveldt, N. , Tijsma, G. , Janssen, T. , & Kupper, F. (2019). Responsible research and innovation as a novel approach to guide educational impact of mind, brain, and education research. Mind, Brain, and Education, 13(4), 279–287. 10.1111/mbe.12213 PMC6867903 31749892

[mbe12250-bib-0021] van Atteveldt, N. , van Kesteren, M. T. , Braams, B. , & Krabbendam, L. (2018). Neuroimaging of learning and development: Improving ecological validity. Frontline Learning Research, 6(3), 186–203. 31799220 10.14786/flr.v6i3.366 PMC6887532

[mbe12250-bib-0022] van der Aar, L. E. , Crone, E. A. , & Peters, S. (2019). What characterizes adolescents struggling with educational decision‐making? The role of behavioral and neural correlates of self‐concept and self‐esteem. Mind, Brain, and Education, 13(3), 184–197.

[mbe12250-bib-0023] Vaughn, A. R. , Brown, R. D. , & Johnson, M. L. (2020). Understanding conceptual change and science learning through educational neuroscience. Mind, Brain, and Education, 14(2), 82–93. 10.1111/mbe.12237

[mbe12250-bib-0024] Vendetti, M. S. , Matlen, B. J. , Richland, L. E. , & Bunge, S. A. (2015). Analogical reasoning in the classroom: Insights from cognitive science. Mind, Brain, and Education, 9(2), 100–106. 10.1111/mbe.12080

[mbe12250-bib-0025] von Spreckelsen, M. , Dove, E. , Coolen, I. , Mills, A. , Dowker, A. , Sylva, K. , … Scerif, G. (2019). Let's talk about maths: The role of observed “maths‐talk” and maths provisions in preschoolers' numeracy. Mind, Brain, and Education, 13(4), 326–340.

[mbe12250-bib-0026] Wu, L. , & Kim, M. (2019). See, touch, and feel: Enhancing young children's empathy learning through a tablet game. Mind, Brain, and Education, 13(4), 341–351.

